# Differential patterns of insulin secretion and sensitivity in patients with type 2 diabetes mellitus and nonalcoholic fatty liver disease versus patients with type 2 diabetes mellitus alone

**DOI:** 10.1186/1476-511X-13-7

**Published:** 2014-01-07

**Authors:** Shang-Yu Chai, Xiao-Yu Pan, Ke-Xiu Song, Yue-Ye Huang, Fei Li, Xiao-Yun Cheng, Shen Qu

**Affiliations:** 1 Department of Endocrinology, Shanghai 10th People’s Hospital, Tongji University, Shanghai, China; 2 Shanghai 10th People’s Hospital, Nanjing Medical University, 301 Middle Yanchang Road, Shanghai, China

**Keywords:** Nonalcoholic fatty liver disease, Impaired glucose tolerance, Type 2 diabetes mellitus, Hyperinsulinemia, Insulin resistance

## Abstract

**Background:**

Nonalcoholic fatty liver disease (NAFLD) and type 2 diabetes mellitus (T2DM) often coexist and have adverse outcomes. The aim of our study was to elucidate metabolic abnormalities in patients with DM-NAFLD versus those with T2DM alone.

**Methods:**

Patients were divided into two groups: 26 T2DM patients with NAFLD and 26 gender-, age-, and body mass index-matched patients with T2DM alone. Patients took a 75-g oral glucose tolerance test (OGTT), which measured serum insulin and C-peptide (C-p) levels at baseline (0 min), 30 min, 60 min, and 120 min after glucose challenge.

**Results:**

Patients with DM-NAFLD or T2DM alone had similar blood glucose levels. β-cell hypersecretion was more obvious in patients with DM-NAFLD. In addition, fasting, early-phase, and late-phase C-peptide levels were significantly increased in patients with DM-NAFLD (ΔC-p 0–30 min, *P* < 0.05; Area Under the Curve (AUC) C-p/PG 30–120 min ratio, *P* < 0.01; and AUC C-p 30–120 min, *P* < 0.01). Hepatic and extrahepatic insulin resistance during the OGTT did not differ significantly between groups. Hepatic insulin sensitivity independently contributed to the early phase (0–30 min) of the OGTT in patients with T2DM and NAFLD, whereas a significant deficit in late insulin secretion independently contributed to the 30–120 min glucose status in patients with T2DM only.

**Conclusions:**

In patients with similar levels of insulin resistance and hyperglycemia, DM-NAFLD was associated with higher serum insulin levels than T2DM alone. Hyperinsulinemia is caused mainly by β-cell hypersecretion. The present study demonstrates pathophysiological differences in mechanisms of insulin resistance in patients with DM-NAFLD versus T2DM alone.

## Background

Nonalcoholic fatty liver disease (NAFLD) and type 2 diabetes mellitus (T2DM) are two of the most common chronic diseases worldwide. Because they share the pathogenic abnormalities of excess adipose tissue and insulin resistance, both NAFLD and T2DM frequently coexist [1], and the adverse outcomes of each can overlap in affected individuals. T2DM is a risk factor for progressive liver disease and liver-related death in patients with NAFLD, and NAFLD may be a marker of increased cardiovascular risk and mortality in individuals with T2DM [1], whereas patients with both NAFLD and T2DM have poorer prognoses in terms of increased cardiovascular and liver-related mortality [2].

Although the pathophysiology of NAFLD and T2DM are intimately linked, evidence from rodent and human models suggests that distinct molecular pathways serve the ability of insulin to promote lipogenesis and suppress gluconeogenesis [3]. Under normal circumstances, insulin stimulation of the insulin receptor (INSR) activates independent arms of the signaling pathway: (1) AKT2 activation, phosphorylation of FOXO1, and inhibition of gluconeogenic gene transcription, leading to decreased glucose output; (2) activation of SREBP-1c-targeted lipogenic genes, leading to secretion of triglyceride (TG)-rich very low density lipoproteins (VLDLs). Patients with INSR mutations or INSR antibodies showed severe hyperglycemia and hyperinsulinemia but normal plasma lipid levels. Mutations in the AKT2 gene impair the inhibition of gluconeogenesis, without abolishing the lipogenic effect of insulin, resulting in increased blood glucose as well as hepatosteatosis and metabolic dyslipidemia, with elevated plasma TG and depressed high-density lipoprotein cholesterol levels [4].

Insulin-resistant patients with liver steatosis, compared with insulin-sensitive individuals, have greater insulin responses and lower hepatic insulin clearance, leading to hyperinsulinemia [5]. T2DM patients with NAFLD could also have different metabolic characteristics when compared with patients with T2DM only. The demonstration of metabolic abnormalities that differ between patients with DM-NAFLD and those with T2DM only may provide the framework for developing effective treatment strategies for both groups of patients.

## Results

The characteristics of the patients are shown in Table 1. Age, gender, and BMI were well matched between patients with and without NAFLD. NAFLD was diagnosed based on the clinical information, and the diagnosis was confirmed using MRS.

**Table 1:** Baseline characteristics of the patients

	**T2DM**	**T2DM + NAFLD**	**P-value**
**Number of patients**	26	26	NS
**Female**	15	16	NS
**Age in years**	54.92	52.38	NS
**BMI (kg/m**^**2**^**)**	28.2 ± 3.7 kg/m^2^	27.2 6 ±3.6 kg/m^2^	NS
**Fat content in the liver (%)**	2 ± 0.01	69.09 ± 0.51	<0.01
**Fasting plasma glucose (mmol/l)**	9.26 ± 2.67	9.15 ± 2.77	NS
**Plasma glucose at 120 min (mmol/l)**	15.65 ± 3.92	15.82 ± 2.86	NS
**Glucose **_**AUC **_**(mmol · 1**^**-1**^ **· h**^**-1**^**)**	1787.71 ± 413.694	1746.75 ± 301.48	NS

Data are means ± SD. Student’s *t*-test (differences between patients with DM-NAFLD and those with T2DM only). Fat content in the liver (%) were measured by magnetic resonance spectroscopy (MRS) and expressed by the ratio of fat/water of the liver.

**Figure 1:**
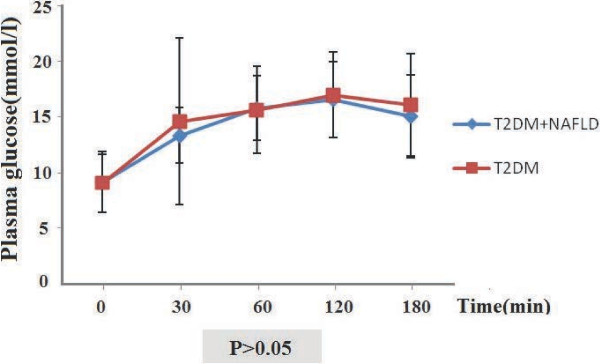
**Plasma glucose during OGTTs performed in patients with DM-NAFLD and those with T2DM only.** Plasma glucose concentrations at 0, 30, 60, 120, and 180 min after the OGTT was administered. P > 0.05 for each timepoint.

Given the different degrees of hyperglycemia in T2DM patients, the plasma glucose curves during the OGTT were initially investigated regarding the pathophysiology of glucose intolerance in patients with DM-NAFLD and those with T2DM only. As shown in Figure 1, the glucose curves were very similar, and the plasma glucose levels were not significantly different between patients with DM-NAFLD and those with T2DM only. There was an initial rapid increase in the plasma glucose concentration between 0 and 30 min, and a gradual increase between 30 and 120 min. The peaks of the glucose levels occurred at 120 min, and then were reduced and remained at a markedly increased level between 120 and 180 min.

**Figure 2:**
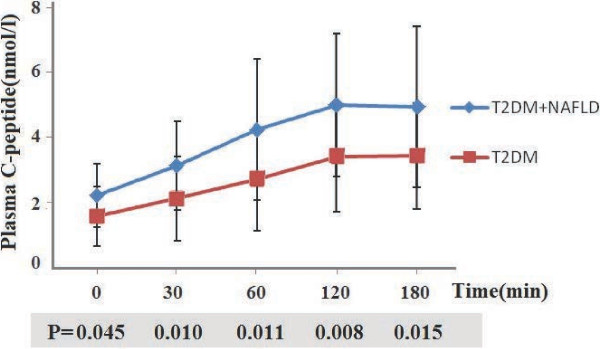
**Plasma C-peptide concentrations during OGTTs performed in patients with DM-NAFLD and those with T2DM only.** Plasma C-peptide concentrations at 0, 30, 60, 120, and 180 min after the OGTT was administered.

The C-peptide concentration curves (Figure 2) had a shape very similar to that of the glucose curves. C-peptide concentrations were significantly higher in patients with DM-NAFLD compared with those with T2DM only, at fasting, and at 30 min, 60 min, and 2 h after glucose challenge (Figure 2).

To investigate the etiology of the different patterns of insulin secretion between the T2DM patients with or without NAFLD, the C-peptide response to glucose load was analyzed (Table 2). Significant C-peptide hypersecretion occurred in patients with DM-NAFLD during the fasting, early, and late phases of the OGTT. This hypersecretion, however, was no longer evident after the fasting C-peptide was normalized to the fasting plasma glucose (FPG). By contrast, following the glucose load and considering early, late, and total β-cell secretion, the ratio of incremental C-peptide to incremental plasma glucose was still significantly higher in patients with DM-NAFLD than in those with T2DM only (ΔC-p/PG 30 min ratio, *P* = 0.011; C-p/PG 120–180 min ratio, *P* = 0.025; Area Under the Curve (AUC) C-p/PG ratio, *P* = 0.007). The insulin resistance index (HOMA-IR) and muscle insulin sensitivity index during the second hour of the OGTT were similar in patients with DM-NAFLD and those with T2DM only.

**Table 2:** Insulin secretion and insulin sensitivity in patients with DM-NAFLD and those with T2DM only

	**T2DM**	**T2DM + NAFLD**	***P*-value**
**Fasting C-peptide (nmol/l)**	1.56 ± 0.9	2.2 ± 0.97	0.045
**2-h OGTT**	3.37 ± 1.66	4.95 ± 2.16	0.008
**ΔC-peptide 30 min (pmol/l)**	0.506 ± 0.9	0.97 ± 0.74	0.045
**ΔC-p/PG 30 min ratio**	0.137 ± 0.1	0.247 ± 0.182	0.011
**AUC C-p (pmol/l)**	323.39 ± 161.13	466.71 ± 208.51	0.007
**AUC C-p/PG ratio**	0.18 ± 0.09	0.27 ± 0.13	0.008
**AUC C-p 30–120 min (pmol/l)**	255.16 ± 128.93	385.9 ± 178.06	0.003
**AUC C-p/PG 30–120 min ratio**	0.186 ± 0.097	0.281 ± 0.139	0.006
**AUC C-p 120–180 min**	105.55 ± 48.21	149.21 ± 67.54	0.009
**AUC C-p/PG 120–180 min**	0.235 ± 0.128	0.619 ± 0.179	0.025
**Muscle insulin sensitivity index**	0.209 ± 1.71	0.34 ± 0.56	NS
**HOMA2-B%**	97.62 ± 50.66	107.33 ± 44.86	NS
**HOMA2-S%**	31.39 ± 20.31	23.65 ± 11.97	NS
**HOMA-IR**	4.44 ± 2.5	5.4 ± 2.9	NS

Data are means ± SD; Δ, incremental; NS, *P* > 0.05 by Student’s *t*-test.

Multiple linear regression analysis was performed to evaluate the impact of hepatic insulin sensitivity and suppression of endogenous glucose production (EGP), peripheral insulin sensitivity in muscle, and the pattern of insulin release on the variability in the OGTT (Table 3). HOMA-IR and HOMA-B was negatively associated with EGP in both patients with DM-NAFLD and patients with T2DM only. A significant positive effect of early-phase insulin sensitivity was associated with the AUC of PG levels at 0–30 min only in patients with DM-NAFLD (*Bate* = 0.625, *P* = 0.001) and a negative effect of insulin secretion was associated with the AUC of PG levels at 30–120 min only in patients with T2DM only (*Bate* = -0.413, *P* = 0.036). The indicators of late phase-insulin secretion showed a similar negative effect on PG 120–180 min levels in both patients with DM-NAFLD and those with T2DM only.

**Table 3:** Multiple linear regression analysis of plasma glucose with each of the dependent variables

		**Fasting**		**AUC PG**		**AUC PG**		**AUC PG**	
		**(PG 0 min)**		**0–30 min**		**30–120 min**		**120–180 min**	
		***Bate***	***Sig***	***Bate***	**Sig**	***Bate***	***Sig***	***Bate***	***Sig***
T2DM + NAFLD	Insulin sensitivity	0.634	0.001*	0.625	0.001*	0.378	0.057	0.042	0.839
	Insulin secretion	-0.545	0.004*	0.118	0.566	-0.355	0.075	-0.570	0.002*
T2DM	Insulin sensitivity	0.413	0.036*	0.099	0.629	0.038	0.855	0.155	0.449
	Insulin secretion	-0.626	0.001*	-0.227	0.265	-0.413	0.036*	-0.657	0.000*

The variation in the OGTT with each of the dependent variables: Insulin sensitivity = HOMA-IR, muscle insulin sensitivity index; Insulin secretion = ΔC-p/PG 30 min ratio, ΔC-p/PG 30 min ratio, AUC C-p/PG 30–120 min ratio, AUC C-p/PG 120–180 min. *, *P *< 0.05.

## Discussion

Although patients with DM-NAFLD or T2DM only showed no difference in age, BMI, FPG, and glucose intolerance, they exhibited significantly different degrees of impairment in insulin secretion and insulin resistance as determined by a 75-g standard OGTT. This study showed that patients with DM-NAFLD had higher plasma C-peptide concentrations than those with T2DM only most likely to suppress EGP and also in response to variation in plasma glucose during the OGTT.

Proposed mechanisms of hyperinsulinemia have been reported by several authors, including increased insulin secretion by the pancreas, diminished hepatic insulin extraction, or a combination of the two. In advanced liver disease, insulin clearance is decreased and is considered one of the main causes of hyperinsulinemia in patients with liver cirrhosis [6-8]. Because fat accumulation in the liver may affect insulin clearance [9], it has long been debated whether impaired insulin clearance in patients with NAFLD is one of the causes of hyperinsulinemia [10]. To investigate true pancreatic insulin secretion, which excludes the effects of insulin extraction by the liver with exogenously administered insulin and insulin sensitizing, we used C-peptide to monitor the average β-cell insulin secretion. C-peptide is secreted by pancreatic β-cells in equal amounts with insulin; however, unlike insulin, C-peptide is not extracted by the liver and has a constant peripheral clearance [11-13]. Thus, plasma C-peptide concentrations may reflect true pancreatic insulin secretion more accurately than the level of plasma insulin itself. Significant β-cell hypersecretion appeared in patients with DM-NAFLD after glucose loading during fasting, early, and late phases based on the total AUC of plasma C-peptide concentrations after the OGTT, compared with non-NAFLD patients who were well matched for baseline glycemic parameters. The β-cell hypersecretion, however, was no longer obvious when the fasting C-peptide was normalized to the FPG. By contrast, following the glucose load, the ratio of incremental C-peptide to incremental plasma glucose levels was still significantly higher in patients with DM-NAFLD than in those with T2DM only. This could be explained by significant additional β-cell insulin secretion in patients with DM-NAFLD following oral glucose administration.

Insulin sensitivity and insulin secretion are reciprocally related. Reduced insulin sensitivity and compensatory hyperinsulinemia play key etiologic roles in the development of NAFLD [14-16]. In our study, hepatic insulin resistance, as calculated by HOMA-IR (derived from FPG and fasting plasma insulin concentrations), was not significantly different between patients with DM-NAFLD and those with T2DM only. The muscle insulin sensitivity index was measured by the rate of decline in plasma glucose concentration divided by the plasma insulin concentration. The decline from the peak plasma glucose concentration during the OGTT primarily reflects glucose uptake by peripheral tissues such as skeletal muscle. This index showed a good correlation with the muscle insulin sensitivity measurement by the euglycemic-hyperinsulinemic clamp in obese patients with normal glucose tolerance (*r* = 0.78, *P* < 0.0001) [17]. The patients with DM-NAFLD and those with T2DM only had similar muscle insulin sensitivities at 120–180 min following the glucose load as plasma glucose levels began to decline.

The present study also examined the difference in the relative contributions of both insulin secretion and insulin sensitivity to the suppression of EGP and variations in oral glucose tolerance in patients with DM-NAFLD and those with T2DM only. A predominantly positive association between HOMA-IR and FPG and a negative association between HOMA-B and FPG were observed in both groups. Patients with DM-NAFLD and those with T2DM only manifested similarly severe defects in late-phase insulin in responses to oral glucose intake. In addition, hepatic insulin sensitivity independently contributed to the early phase (0–30 min) of the OGTT in patients with T2DM and NAFLD, whereas a significant deficit in late-phase insulin secretion independently contributed to glucose disposal into peripheral tissues at 30–120 min during the OGTT in the T2DM-only group. This could be explained by the different postreceptor defects leading to insulin resistance and the different patterns of impaired insulin secretion between patients with DM-NAFLD and those with T2DM only.

Our data suggest that relative hyperinsulinemia is characteristic of patients with DM-NAFLD compared with those with T2DM only. Hyperinsulinemia in patients with DM-NAFLD is caused primarily by β-cell hypersecretion in response to a similar degree of insulin resistance and the equivalent level of hyperglycemia in patients with T2DM only during the OGTT. This distinct difference in the underlying pathophysiology of patients with DM-NAFLD and those with T2DM only may have consequences for clinical outcomes. Clearly, there are limitations that hinder adjusting the insulin dose by measuring the plasma glucose value alone. Exogenously administered insulin does not mimic endogenous insulin secretion [18]. Thus, therapeutically increasing insulin doses may result in increased peripheral hyperinsulinemia in patients with DM-NAFLD, further increasing the risk of hypoglycemia. Additionally, studies have shown that insulin directly promotes fat accumulation in liver cells, further contributing to nonalcoholic steatohepatitis, which is an increasingly frequent cause of cirrhosis, hepatocellular carcinoma, and liver failure [19,20]. Increasing exogenously administered insulin doses results in further peripheral hyperinsulinemia, which may worsen NAFLD.

## Conclusions

In patients with similar levels of insulin resistance and hyperglycemia, DM-NAFLD was associated with higher serum insulin levels than T2DM alone. Hyperinsulinemia is caused mainly by β-cell hypersecretion. The results described herein highlight the key pathophysiological differences distinguishing patients with DM-NAFLD compared with those with T2DM only and demonstrate the critical role of hyperinsulinemia in the disease process. There remains a need for new interventions for delaying or preventing the onset of NAFLD, which should not only suppress hepatic gluconeogenesis but also aim to reduce patients’ plasma insulin levels.

## Methods

### Patients

The study was approved by the institutional review board of the Shanghai 10^th^ People’s Hospital of Tongji University. Written informed consent was obtained from all study participants. A total of 52 Chinese patients with T2DM who met American Diabetes Association diagnostic criteria (fasting glucose >7.0 mmol/l and 2-h glucose >11.1 mmol/l) [21] and had NAFLD (n = 26) were recruited or invited to participate in the study. Both groups were matched for gender, age, and body mass index (BMI). The patients with and without NAFLD were diagnosed by magnetic resonance spectroscopy (MRS). The exclusion criteria were as follows: *1*) alcohol consumption >20 g/day; and *2*) no serological evidence of autoimmune hepatitis or hepatitis A, B, or C, or clinical signs or symptoms of inborn errors of metabolism or a history of using toxins or drugs known to induce hepatitis. All the study participants were ethnic Han Chinese.

### Oral glucose tolerance test (OGTT)

After an overnight fast for 10 h, an indwelling catheter was inserted into a forearm vein, and blood samples were obtained 15 min before the 75-g OGTT, and then at intervals of 0, 30, 60, 120, and 180 min for the measurement of plasma glucose and C-peptide (C-p) concentrations.

### C-p, insulin, and glucose assays

Blood was collected into fluoride oxalate-containing tubes for measurement of glucose (YSI 2300; YSI, Hants, UK) and lithium-heparin-containing tubes for measurement of C-peptide levels. The within- and between-assay coefficients of variation were 5.4% and 8.8%, respectively, for the C-peptide assay and 4.1% and 8.8%, respectively, for the insulin assay.

### Calculations

We measured C-peptide concentrations during the OGTT at the following timepoints: fasting plasma C-peptide concentration (C-p 0 min), early-phase plasma C-peptide concentration (C-p 0–30 min), plasma C-p concentration (C-p 30–120 min), and late-phase plasma C-peptide concentration (C-p 120–180 min), based on the plasma glucose curve of the patients during the OGTT [17]. As indicators of hepatic insulin resistance, we used the homeostasis model assessment (HOMA) insulin resistance index (HOMA-IR) [22]. The muscle insulin sensitivity index used in the study was defined as dG/dt divided by the mean C-peptide concentration during the OGTT, where dG/dt is the rate of decline in plasma glucose concentration and is calculated as the slope of the least square fit to the decline in plasma glucose concentration from peak to nadir. The areas under the curve were calculated by the trapezoidal rule for each of the time periods [23,24].

### Statistical analysis

All data are presented as means ± standard deviation (SD). For comparison between groups, Student’s *t*-test was used. To compare the mean of more than two groups, one-way analysis of variance (ANOVA) was used. Multiple stepwise linear regression analysis was used to study the independent influence of insulin response and hepatic and extrahepatic insulin sensitivity on the plasma glucose variability during the OGTT. Results were considered statistically significant at *P* value less than 0.05.

## Abbreviations

AUC: Area under the curve; FPG: Fasting plasma glucose; HOMA: Homeostasis model assessment; HOMA-IR: HOMA, Insulin resistance index; HOMA-B: HOMA, Insulin secretion index; C-p: C-peptide; NAFLD: Nonalcoholic fatty liver disease; T2DM: Type 2 diabetes mellitus; EGP: Endogenous glucose production.

## Competing interests

The authors declare that they have no competing interests.

## Authors’ contributions

SC and SQ conceived of the study, and participated in its design and coordination and helped to draft the manuscript. XP, YH, KS, FL,XC participated in the design of the study and performed the statistical analysis. All authors read and approved the final manuscript.

## References

[B1] Smith BW Adams LA Nonalcoholic fatty liver disease and diabetes mellitus: pathogenesis and treatment Nat Rev Endocrinol 2011 7 456 465 10.1038/nrendo.2011.72 21556019

[B2] Semple RK Sleigh A Murgatroyd PR Adams CA Bluck L Jackson S Vottero A Kanabar D Charlton-Menys V Durrington P Postreceptor insulin resistance contributes to human dyslipidemia and hepatic steatosis J Clin Invest 2009 119 315 322 19164855 10.1172/JCI37432 PMC2631303

[B3] Hegele RA Reue K Hoofbeats, zebras, and insights into insulin resistance J Clin Invest 2009 119 249 251 19244606 10.1172/JCI38420 PMC2631308

[B4] Cohen JC Horton JD Hobbs HH Human fatty liver disease: Old questions and New insights Science 2011 332 1519 1523 10.1126/science.1204265 21700865 PMC3229276

[B5] Kotronen A Vehkavaara S Seppala-Lindroos A Bergholm R Yki-Jarvinen H Effect of liver fat on insulin clearance Am J Physiol Endocrinol Metab 2007 293 E1709 E1715 10.1152/ajpendo.00444.2007 17895288

[B6] Kruszynska YT Goulas S Wollen N McIntyre N Insulin secretory capacity and the regulation of glucagon secretion in diabetic and non-diabetic alcoholic cirrhotic patients J Hepatol 1998 28 280 291 10.1016/0168-8278(88)80015-1 9514541

[B7] Kaji K Yoshiji H Kitade M Ikenaka Y Noguchi R Yoshii J Yanase K Namisaki T Yamazaki M Moriya K Impact of insulin resistance on the progression of chronic liver diseases Int J Mol Med 2008 22 801 808 19020779

[B8] Goto T Onuma T Takebe K Kral JG The influence of fatty liver on insulin clearance and insulin resistance in non-diabetic Japanese subjects Int J Obes Relat Metab Disord 1995 19 841 845 8963349

[B9] Kotronen A Juurinen L Tiikkainen M Vehkavaara S Yki-Jarvinen H Increased liver fat, impaired insulin clearance, and hepatic and adipose tissue insulin resistance in type 2 diabetes Gastroenterology 2008 135 122 130 10.1053/j.gastro.2008.03.021 18474251

[B10] Alibegovic AC Hojbjerre L Sonne MP van Hall G Stallknecht B Dela F Vaag A Impact of 9 days of bed rest on hepatic and peripheral insulin action, insulin secretion, and whole-body lipolysis in healthy young male offspring of patients with type 2 diabetes Diabetes 2009 58 2749 2756 10.2337/db09-0369 19720789 PMC2780872

[B11] Brons C Jensen CB Storgaard H Hiscock NJ White A Appel JS Jacobsen S Nilsson E Larsen CM Astrup A Impact of short-term high-fat feeding on glucose and insulin metabolism in young healthy men J Physiol-London 2009 587 2387 2397 10.1113/jphysiol.2009.169078 19332493 PMC2697306

[B12] Marx N Walcher D C-peptide and atherogenesis: C-peptide as a mediator of lesion development in patients with type 2 diabetes mellitus? Exp Diabetes Res 2008 2008 385108 18401446 10.1155/2008/385108 PMC2288642

[B13] Kim CH Park JY Lee KU Kim JH Kim HK Association of serum gamma-glutamyltransferase and alanine aminotransferase activities with risk of type 2 diabetes mellitus independent of fatty liver Diabetes Metabol Res Rev 2009 25 64 69 10.1002/dmrr.890 19065605

[B14] Bugianesi E McCullough AJ Marchesini G Insulin resistance: a metabolic pathway to chronic liver disease Hepatology 2005 42 987 1000 10.1002/hep.20920 16250043

[B15] Haque M Sanyal AJ The metabolic abnormalities associated with non-alcoholic fatty liver disease Best Pract Res Clin Gastroenterol 2002 16 709 731 10.1053/bega.2002.0325 12406441

[B16] Marchesini G Brizi M Bianchi G Tomassetti S Bugianesi E Lenzi M McCullough AJ Natale S Forlani G Melchionda N Nonalcoholic fatty liver disease - a feature of the metabolic syndrome Diabetes 2001 50 1844 1850 10.2337/diabetes.50.8.1844 11473047

[B17] Matsuda M DeFronzo RA Insulin sensitivity indices obtained from oral glucose tolerance testing - comparison with the euglycemic insulin clamp Diabetes Care 1999 22 1462 1470 10.2337/diacare.22.9.1462 10480510

[B18] Liou I Kowdley KV Natural history of nonalcoholic steatohepatitis J Clin Gastroenterol 2006 40 S11 S16 16540761 10.1097/01.mcg.0000168644.23697.31

[B19] Farrell GC Larter CZ Nonalcoholic fatty liver disease: from steatosis to cirrhosis Hepatology 2006 43 S99 S112 10.1002/hep.20973 16447287

[B20] Brown MS Goldstein JL Selective versus total insulin resistance: a pathogenic paradox Cell Metab 2008 7 95 96 10.1016/j.cmet.2007.12.009 18249166

[B21] Abdul-Ghani MA Matsuda M Balas B DeFronzo RA Muscle and liver insulin resistance indexes derived from the oral glucose tolerance test Diabetes Care 2007 30 89 94 10.2337/dc06-1519 17192339

[B22] Bacha F Gungor N Arslanian SA Measures of beta-cell function during the oral glucose tolerance test, liquid mixed-meal test, and hyperglycemic clamp test J Pediatr 2008 152 618 621 10.1016/j.jpeds.2007.11.044 18410762 PMC2396878

[B23] Potteiger JA Jacobsen DJ Donnelly JE A comparison of methods for analyzing glucose and insulin areas under the curve following nine months of exercise in overweight adults Int J Obes 2002 26 87 89 10.1038/sj.ijo.0801839 11791151

[B24] Greco AV Mingrone G Mari A Capristo E Manco M Gasbarrini G Mechanisms of hyperinsulinaemia in Child’s disease grade B liver cirrhosis investigated in free living conditions Gut 2002 51 870 875 10.1136/gut.51.6.870 12427792 PMC1773476

